# Moral Distress and the Governance of Attention in Nursing: A Hanian Reading of the Structural Restriction of Moral Agency

**DOI:** 10.1111/nup.70100

**Published:** 2026-07-16

**Authors:** Juan Ignacio Rico Becerra, Aarón Muñoz Devesa

**Affiliations:** ^1^ Department of Social and Healthcare Services, Faculty of Health and Social Sciences University of Murcia Lorca Spain

**Keywords:** alterity, Byung‐Chul Han, care ethics, governance of attention, hermeneutics, moral agency, moral distress

## Abstract

Contemporary nursing is increasingly shaped by performance pressures, demands for traceability, and technological mediation. These developments do more than add tasks to everyday clinical work. They also shape what nurses can attend to, how they judge situations, and whether they are able to sustain the care they believe a patient needs. This article examines moral distress as a structural restriction of moral agency: nurses may recognise what a situation requires yet find themselves unable to act accordingly. Drawing on Byung‐Chul Han's analyses of performance, psychopolitics, infocracy, and the palliative society, we develop a tetradic matrix that relates these contemporary logics to four goods of nursing care: attention, clinical judgement, alterity, and hope. In practice, these goods refer to sustained presence, prudent deliberation, recognition of the patient as a singular person, and hope understood as non‐abandonment. Applied to the nursing metaparadigm, the matrix shows how clinical time, attention, documentation, and technological mediation can become places where moral agency is either supported or weakened. The article therefore argues for a shift in the ethical evaluation of nursing work: from a narrow focus on individual resilience towards the governance of attention and the institutional conditions that make responsible care possible.

## 1 Introduction

Recent empirical literature confirms that nurse burnout is a global phenomenon with implications for safety, satisfaction, and quality of care, and that understanding it requires that it not be reduced to an individual problem (Getie et al. 2025; Jun et al. 2021; Li et al. 2024). The specific focus of this article, however, is moral distress in nursing, associated with moral residue, erosion of integrity, and institutional constraints that render the care judged to be due impracticable (Salari et al. 2022; Epstein and Hamric 2009). Although studies of moral distress describe the phenomenon and its consequences convincingly, they have devoted less attention to its embeddedness in contemporary regimes that govern work and to how it is institutionally enacted within the current organisation of care. Under conditions of performance pressure, traceability, and technological mediation, moral distress in nursing may be read as a structural restriction of moral agency. Nurses may know what care requires and still be unable to provide it. In practice, this can mean being unable to assess, deliberate, or sustain the presence that a particular case calls for (Han 2012, 2021a). Responses focused only on individual resilience are therefore inadequate, since even competent and resilient nurses may experience moral distress when organisational conditions prevent them from sustaining the care they judge to be due.

Healthcare digitalisation, the expansion of the electronic health record (EHR), and indicator‐based governance have intensified the technological mediation of care. Documentary traceability, continuous audit, and digital communication do more than add new tasks; they reorganise priorities and the temporal structure of clinical work. Shifts are divided into recordable micro‐actions, while attention is dispersed across streams of notifications. Under these conditions, the organisation of work comes to shape what is recognised as good practice and what falls outside the field of visibility (Doherty et al. 2025; World Health Organisation 2021b; McBride et al. 2018). This is one way in which technologically mediated care can erode relational moral agency at a structural level. For example, a completed fall‐risk template may be more visible to the organisation than the unhurried conversation through which a nurse understands why a disoriented patient keeps trying to leave the bed. The first action is easily recorded and audited; the second may be ethically decisive, but institutionally less visible if the system recognises only what can be entered into predefined fields.

In this scenario, Jameton and subsequent developments in the literature on moral distress provide an indispensable vocabulary for thinking about structural limits, value conflicts, moral residue, and eroded integrity (Jameton 1984; Morley and Sankary 2024; Manara et al. 2014; Hardingham 2004). At the same time, a theory analysis of moral distress has suggested that earlier formulations suffered from limited theoretical clarity and from an insufficient account of their relational and agency‐centred dimensions (Wilson 2018). Our argument relocates this problem within an economy of attention that consumes clinical time, increases documentary burden, and presses for the rapid closure of suffering (Han 2012, 2021b, 2022, 2021c). What Han contributes here is a philosophical grammar for naming four contemporary modes of governing work—performance, psychopolitics, infocracy, and the palliative society—and for rethinking moral distress as a structural restriction of moral agency rather than as a solely individual difficulty. From this starting point, we propose four structural tensions—performance–attention, infocracy–judgement, psychopolitics–alterity, and palliative society–hope—to analyse how certain forms of institutional organisation erode goods of care without which nursing practice loses its ethical integrity (Tronto 2013; Laurin and Martin 2022; International Council of Nurses 2021; Travelbee 1971).

## 2 Theoretical Framework

When organisational arrangements absorb clinical time and fragment attention, they also narrow the practical space in which moral action can take place. Attention is not treated here as a purely individual capacity, but as something shaped by institutional conditions such as workflows, documentation, alarms, indicators, staffing, continuity, and ethical support. In this sense, the term *governance of attention* refers to the ways institutions and technologies shape what can be attended to, for how long, and with what degree of continuity and depth (Doherty et al. 2025; McBride et al. 2018; Tronto 2013; International Council of Nurses 2021). Moral distress can then be read as a difficulty in sustaining moral agency under conditions of interdependence, vulnerability, and institutional mediation: recognising what care requires, deliberating well, and carrying that care through in practice (Jameton 1984; Morley and Sankary 2024; Musto and Rodney 2016; Hardingham 2004; Travelbee 1971). In practice, this can mean being unable to attend with continuity, to judge with the necessary room for prudent deliberation, or to respond to the other in the way that their situation requires, in light of relationships, shared vulnerability, and the organisational conditions that make such moral exercise possible—or prevent it (Morley and Sankary 2024; Tronto 2013; Travelbee 1971).

This argument requires a conceptual vocabulary that is philosophical in origin but clinically focused in use. By a hermeneutic approach, we mean an interpretive reading that translates philosophical categories into clinical and organisational questions. Han's concepts are therefore used in a deliberately narrow sense. In this article, infocracy points to the way data, audit, and traceability can become signs of organisational legitimacy. Psychopolitics is used to describe how institutional demands may be internalised by professionals as self‐demand. The palliative society helps name the tendency to neutralise suffering or press it towards rapid resolution. Alterity, finally, marks the recognition of the patient as a singular other, not simply as a case, profile, or task. Read in this way, these terms do not function as abstract labels added to nursing practice from outside. They help name how concrete organisational arrangements can restrict attention, judgement, relational responsibility, and the capacity to sustain care.

### 2.1 Tetradic Logic of Tensions

We propose an original theoretical‐conceptual matrix for analysing the contemporary crisis of nursing work as a clash between modes of organising work and goods of care without which practice loses its ethical integrity. Within this framework, ethical tension refers to situations in which institutional design renders central capacities of nursing practice impracticable: attending, judging prudently, recognising alterity, and sustaining hope. Its analytical value lies in making it possible to trace the movement from concrete organisational mechanisms to forms of moral damage and restriction of agency, and from there to institutional obligations and prudential indicators for the governance of attention.

Within this line of analysis, Byung‐Chul Han's diagnoses provide concepts for identifying tendencies that, in clinical settings, take material form in concrete organisational mechanisms. The disciplinary pole of this matrix shows why these are ethically significant: such goods are understood as conditions without which care is reduced to technical‐administrative execution. In clinical terms, this means losing sustained presence, room for prudent deliberation, the capacity to recognise the other as a singular person, and the possibility of sustaining non‐abandonment. Read through the lens of virtue ethics, this dimension may be understood in terms of the internal goods of nursing practice, constitutive of a cooperative activity oriented towards vulnerable and interdependent subjects (Tronto 2013, 1993; MacIntyre 2001). Their selection is strategic rather than exhaustive. Within this framework, these goods are prioritised because they bring into focus core conditions for ethical care that are especially vulnerable under contemporary forms of work organisation.

Taken together, the framework links these contemporary diagnoses with the disciplinary goods, the metaparadigmatic domains, and their institutional implications. As the diagnoses have already been outlined, the next section focuses on the disciplinary goods of nursing care. Their conceptual grounding is developed in the main text.

### 2.2 Disciplinary Goods of Nursing Care

#### 2.2.1 Attention

Following Tronto, attention refers to the capacity to recognise needs and respond to them. In nursing care, it depends on continuity, limited interruption, and sufficient room for prudent deliberation. Read through Han; it also has a contemplative dimension: the possibility of pausing, dwelling, and understanding without reducing the person in advance to data, protocol, or metric. For that reason, protecting attention becomes a matter of institutional design (Tronto 2013; Han 2023b).

#### 2.2.2 Judgement

Nursing judgement integrates data, experience, and context in situations that are not reducible to an algorithm; it requires unfragmented temporality and an orientation towards clinical meaning. Its erosion appears when traceability and protocolisation displace prudential deliberation and make what is correct equivalent to what is documented (Benner 1984; Han 2023a; Doherty et al. 2025).

#### 2.2.3 Alterity

Alterity affirms the primacy of the other as a source of moral obligation. It is eroded when the person is treated as a case, profile, or management unit and the encounter loses ethical density. In nursing care, this dimension protects singularity against the administrative‐technological reduction of the patient and requires conditions for listening, interpreting, and responding to a concrete person, so that the clinical encounter retains primacy in the organisation of work (Levinas 2020; Martinsen 2006).

#### 2.2.4 Hope

Clinical hope is not equivalent to optimism. It denotes a practice of presence and non‐abandonment that sustains meaning and possibility under adverse conditions, without denying suffering or promising total control. It depends on continuity, time, and an institutional culture that does not expel pain; in existential terms, it may be understood as a task in the face of despair and, in nursing terms, as a relational component of clinical accompaniment that helps sustain orientation, presence, and continuity even when there is no immediate resolution (Travelbee 1971; Lohne 2022; Kierkegaard 2008). For that reason, hope is treated here as a distinct disciplinary good: it names not only accompaniment itself, but the practical orientation that allows meaning and clinical possibility to be sustained where there is no resolution, control, or closure of suffering.

### 2.3 Analytical Procedure

This matrix is operationalised through an interpretive procedure at three levels: (1) semantic stabilisation of contemporary diagnoses, (2) translation into disciplinary goods of care, and (3) anchoring in concrete organisational mechanisms. Here, translation refers to an operation of re‐reading and re‐anchoring, rather than to a mechanical equivalence between philosophical categories and clinical phenomena or a linear deduction from a general theory to practice. It involves reinterpreting these diagnoses from the ontology of care in order to specify what forms of work governance they name, what clinical experiences they render visible, and which conditions of possibility for ethical care are thereby compromised. In practical terms, this procedure does not ask the reader to accept Han's concepts as clinical facts; it uses them as lenses through which familiar organisational experiences can be named, compared, and ethically examined.

Normative derivation constitutes the final step; if a disciplinary good functions as a condition of possibility for ethical care, its structural blockage generates institutional obligations of correction oriented towards dignity (Gastmans 2013). For each metaparadigmatic domain, the procedure follows the same analytical sequence: domain, diagnosis, threatened good, dominant organisational mechanism, form of moral damage or restriction of agency, and guiding question. The table serves an expository and heuristic function. It sets out this progression and indicates how the philosophical reading is translated into institutional moral discernment. Its categories should therefore be read in context rather than treated as fixed measures or as a closed managerial device.

### 2.4 Deployment of the Matrix

The analysis is organised around four dominant tensions. This is a preferential rather than exclusive assignment. Each contemporary logic may erode several goods of care, but the framework identifies the pairing in which the governing mechanism most directly affects the good at stake. The criterion is not one of causal exclusivity, but of analytical primacy: in each case, the selected relationship is the one in which the collision between organisational logic and disciplinary good is most visible and clinically recognisable. Thus, for example, infocracy primarily threatens judgement by displacing interpretive deliberation under the primacy of what is traceable and measurable, even though it may also fragment attention. On this basis, the dominant tensions are set out below. The following pairings should therefore be read as dominant emphases, not as mutually exclusive categories. In practical terms, the same EHR alert may belong analytically to infocracy when it subordinates judgement to documentation, and to performance when it interrupts the continuity of attention. The point is not to eliminate this overlap, but to indicate which aspect of the situation is ethically most salient in each case.

#### 2.4.1 Performance–Attention

Under regimes of acceleration, multitasking, and constant availability, nursing attention tends to become reactive: what is attended to is what is urgent and recordable, while sustained presence becomes residual (Han 2012). Restriction of agency appears when what is essential to care is systematically displaced (Morley and Sankary 2024; Hardingham 2004).

#### 2.4.2 Infocracy–Judgement

When documentary traceability, audit, and performance indicators come to organise clinical practice, nursing judgement may be displaced by the primacy of what is traceable and measurable (Han 2022). In clinical settings, documentary expansion may displace prudential deliberation: what is correct becomes confused with what is documented, and what is relevant with what is measurable, so that judgement ends up subordinated to documentary compliance (Doherty et al. 2025; Cho et al. 2024; Gesner et al. 2022).

#### 2.4.3 Psychopolitics–Alterity

Under conditions in which performance demands are internalised as self‐demand and are expressed in the objectification of the patient and the task‐based management of work, professionals may come to govern themselves under a functional logic that reduces the patient to a case or profile. In clinical practice, this logic weakens the ethical encounter and relational responsibility by reorienting attention away from the singularity of the other and towards the functional completion of tasks (Han 2021b; Levinas 2020; Martinsen 2006).

#### 2.4.4 Palliative Society–Hope

Where suffering is treated as something that must be resolved quickly and it becomes institutionally difficult to sustain the limit, hope risks being trivialised as optimism or obligatory positivity. The threatened good is not the expectation of cure, but the capacity to sustain presence, meaning, and non‐abandonment in the face of pain, limit, and finitude (Han 2021c; Travelbee 1971; Lohne 2022; Antunes et al. 2023; Kierkegaard 2008).

### 2.5 Translation Into the Nursing Metaparadigm (Application)

This section organises the analysis by the domains of the nursing metaparadigm and shows, in each case, the articulation between domain, diagnosis, threatened good, dominant mechanism, moral damage or restriction of agency, and guiding question. The nursing metaparadigm is used here as a disciplinary framework for organising the analysis, not as a complete representation of nursing as a discipline. Each domain gives rise to a corresponding institutional obligation. The sequence Person → Environment → Health → Care follows the order of the argument: first the moral subject, then the organisational field that shapes judgement, then the horizon of suffering, and finally the praxis of care. The Environment domain is developed as an illustrative case in order to show how this translation works in practice. The point is not to impose full symmetry across the four domains, but to indicate the rule of translation and the different ways in which it can operate. Table 1 functions as a reading map for this progression and outlines the analytical sequence that follows. It also supports moral discernment within the proposed hermeneutic framework by condensing the conceptual movement that guides the analysis.

**Table 1:** Reading Map for the application of the matrix to the nursing metaparadigm.

Domain	Diagnosis	Threatened good	Dominant mechanism	Moral damage/restriction of agency	Guiding question
Person	Psychopolitics	Alterity	Self‐demand, objectification of the patient, and task‐based management of work	Depersonalisation of the relationship and weakening of relational responsibility	Which organisational conditions contribute to a failure to recognise the person receiving care as a unique individual?
Environment	Infocracy	Judgement	Documentary expansion, audit, and over‐protocolisation driven by performance indicators	Replacement of what is relevant by what is measurable and degradation of clinical judgement	How does the informational environment reorganise the conditions of prudent nursing judgement?
Health	Palliative society	Hope	Pressure to resolve suffering and denial of limits	Difficulty in sustaining non‐abandonment and reduction of hope to optimism	Which institutional conditions make it possible to sustain hope without denying suffering or imposing premature closure?
Care	Performance regime	Attention	Acceleration, multitasking, and pressure for patient flow	Reactivity, fragmentation, and loss of clinical presence	How does the organisation of work affect the possibility of sustaining continuous and morally meaningful clinical attention?

*Note:* The table maps the analytical sequence through which the matrix is applied to the nursing metaparadigm across the domains of person, environment, health, and care, thereby supporting moral discernment within the proposed hermeneutic framework.

#### 2.5.1 Person (Psychopolitics–Alterity)

When the logic of performance is internalised, structural failures such as overload, discontinuity, or documentary pressure tend to be reinterpreted as personal insufficiencies: ‘I cannot keep up’, ‘I am not good enough’. At the same time, the person receiving care risks being objectified and reduced to a case, profile, or management unit. The good that is eroded is alterity, understood as responsibility towards the singularity of the other. The typical mechanism is the internalisation of performance demands, visible in self‐imposed demands, the objectification of the patient, and the task‐based management of work. Moral damage appears when the relationship ceases to operate as a source of obligation and instead becomes organised as a functional transaction, with guilt, moral residue, and eroded professional integrity (Hardingham 2004; Cribb 2011; Han 2021b; Levinas 2020; Martinsen 2006). Within the nursing tradition, Watson and Swanson help clarify what is at stake here: ‘knowing’, ‘being with’, and ‘enabling’ are not secondary affective features, but integral acts of care. When institutional environments normalise their displacement by documentation or technological mediation, and in doing so weaken the conditions for clinical presence, the result is not simply a thinner relational climate but a more basic institutional failure of care (Watson 2008; Sharkey and Sharkey 2012; Swanson 1991). Organisations must therefore protect forms of clinical encounter that cannot be reduced to record‐keeping, including relational continuity and time for presence.

Environment (infocracy–judgement): an illustrative case of translation. In this domain, the analytical sequence becomes especially clear. The governing diagnosis is infocracy, understood here as a mode of organisation that privileges traceability, audit, comparability, and performance indicators as signs of institutional legitimacy. What is most vulnerable under these conditions is clinical judgement, since prudent deliberation requires time, context, and room to integrate data, experience, and the singularity of the case (Benner 1984; Han 2023a). In practice, this logic takes shape through documentary expansion, audit, and forms of over‐protocolisation driven by performance indicators. In the clinical information environment, it is materialised in EHRs, audit‐oriented templates, alerts, messaging, interruptions, and other documentary demands that fragment the temporality of the shift (Lewandowska et al. 2020; Ruppel et al. 2023) and redirect attention towards what can be recorded (Doherty et al. 2025; Cho et al. 2024). Moral damage appears when the nurse recognises that good judgement requires interpretation and deliberation, but the system primarily demands documentation, validation, and responsiveness to interruptions. Under these conditions, what is correct becomes confused with what is documented, and what is relevant with what is measurable. Clinical judgement is thereby weakened, since interpretive nursing knowledge is difficult to recognise when it is not fully traceable. The corresponding institutional response is not to ask individuals to cope better, but to redesign the environment through proportionate documentation, governance of interruptions and alarms, and ethical evaluation of the impact of metrics and technologies on clinical time (World Health Organisation 2021a; American Nurses Association 2022).

#### 2.5.2 Health (Palliative Society–Hope)

When pain is institutionally treated as something to be resolved quickly, health tends to be approached in terms of the reduction of suffering rather than the accompaniment of vulnerability. Clinical hope is at stake here, understood as accompaniment that sustains presence, meaning, and possibility even when cure is not available (Travelbee 1971; Lohne 2022; Antunes et al. 2023). This logic is visible where suffering is pressed towards resolution and the limit becomes difficult to sustain, whether by accelerating relief or by turning distress into a technical problem for immediate management. Moral damage appears when the nurse recognises the need to remain, accompany, and allow time for limit, but the environment rewards closure, positivity, and emotional efficiency (Han 2021c). This makes non‐abandonment harder to sustain and reduces hope to optimism. The institutional response is to guarantee continuity, minimum time for communication, and non‐punitive ethical support that allows limit to be inhabited without cynicism or moral anaesthesia. Within the nursing metaparadigm, health includes biomedical outcomes, but it also concerns the institutional way of inhabiting suffering, vulnerability, and finitude. In this sense, hope remains a relational condition of care where not everything can be resolved at once (Travelbee 1971).

#### 2.5.3 Care (Performance–Attention)

In the praxis of care, the regime of performance puts sustained attention under pressure by turning it into reactive multitasking. This is visible in the acceleration of the shift, pressure for patient flow, and the prioritisation of recordable micro‐tasks (Han 2012; Doherty et al. 2025; Tronto 2013, 1993; Cho et al. 2024). The same logic is intensified when forms of automation redirect clinical time towards validation, classification, and the supervision of system outputs (World Health Organisation 2021a; American Nurses Association 2022). Moral damage appears when nurses recognise the need to be with, deliberate, and accompany, but the organisation of work leaves little room for presence, interpretive pause, and continuity, producing reactivity, fragmentation, and loss of clinical presence. Attention, then, should be understood as an organisational good rather than as an individual virtue of endurance. The institutional response is to redesign workloads, protect continuity, limit destructive multitasking, and secure conditions for a clinical presence that is not merely reactive. This also requires pauses, continuity, and interpretive latitude; without them, attention becomes reactive, clinical judgement deteriorates, and care is impoverished.

## 3 Discussion

Moral distress can be reconsidered here in relation to the organisational conditions under which moral agency is exercised. From this perspective, it points not simply to individual malaise, but to situations in which the organisation of work obstructs the conditions required for ethical nursing care to be sustained (Jameton 1984; Morley and Sankary 2024). Attention, judgement, alterity, and hope matter in this sense as goods internal to practice and as relational goods of care whose preservation depends on institutional conditions (MacIntyre 2001; Tronto 1993). In nursing terms, this concerns the protection of clinical presence, prudent deliberation, the encounter with the singular person, and the possibility of sustaining non‐abandonment. The question, then, is which organisational conditions erode these possibilities, and which help sustain them within clinical practice and institutional design.

The examples developed in this article come largely from hospital‐based and digitally mediated care, but the argument is not intended to confine nursing to those settings. Home nursing brings the same problem into view in a different register. There, pressure may appear in the length of the visit, the route planned for the day, remote monitoring, documentation completed after the encounter, or fragile continuity among professionals, patients, and families. Moral agency is then restricted less by alarm fatigue or patient flow than by the limited room to remain with a person, listen carefully, and judge what the situation requires. The framework can therefore travel beyond institutional settings, but it should not be transferred unchanged; home nursing needs to be examined within its own organisational and relational conditions (Jakobsen et al. 2025).

This also makes it possible to question a recurrent tendency in the applied literature and in organisational responses to treat moral damage mainly as a matter of individual resilience. Such responses may help, but they are insufficient when they take the place of organisational reform and turn institutional failure into a demand for subjective adaptation. At that point, the psychopolitical logic criticised in this article reappears: the system intensifies demands, while the moral cost is reinscribed as individual guilt, thereby obscuring its structural origin (Han 2021b). The point becomes clearer at the level of work organisation. When metrics, documentation, flows, or technologies no longer support clinical judgement but begin instead to govern it, they produce a form of structural moral damage (Doherty et al. 2025; Cho et al. 2024; Gesner et al. 2022). The same occurs when alarms no longer serve as prudent supports for vigilance but instead capture attention, fragment clinical temporality, and reorder the priorities of care (Lewandowska et al. 2020; Ruppel et al. 2023). The ethical issue lies, therefore, in how organisational design orders the relationship between data and the clinical encounter. Data and traceability should remain subordinate to clinical judgement and care, not the other way around (Laurin and Martin 2022; International Council of Nurses 2021).

A first objection should be acknowledged here. Institutional measurement is not always reductive. Some prudential signals may help protect goods of care by making visible fragmentation, documentary overload, discontinuity, or deterioration in deliberation (Newham 2021; Doherty et al. 2025; Cho et al. 2024). Table 2 is intended in this sense: not as a closed evaluative device, but as an illustrative translation into organisational observation and possible signs that institutional design may be either eroding or protecting the basic conditions of ethical care (Laurin and Martin 2022; International Council of Nurses 2021).

**Table 2:** Governance of attention in clinical nursing: mechanisms, moral damage, institutional obligations, and illustrative guides for moral discernment.

Mechanism	Moral damage	Institutional obligation	Illustrative prudential signal
Expansion of EHR‐related work: duplication of data, audit‐oriented templates, checklist logic	Loss of clinical time; deterioration of interpretation, deliberation, and presence; calculative care	Ensure proportionate, non‐redundant, and clinically meaningful documentation	Net documentary burden per shift (minutes or % of shift spent in the EHR; proportion that is redundant)
Environment of interruptions: alerts, messaging, calls, and ad hoc demands	Fragmentation of attention and judgement; increased distress and risk of error	Govern interruptions and protect zones of sustained clinical attention	Attentional fragmentation index (interruptions/hour; % non‐critical)
Alarm overload or poor calibration	Alarm fatigue; deterioration of vigilance, judgement, and moral confidence	Implement alarm governance and reduce non‐actionable alarms	Alarm density and actionability (alarms/bed‐hour; % actionable)
Chronic understaffing and discontinuity	Imposed compensations; reduction of care to performance; degradation of relational continuity	Ensure sufficient staffing and continuity; do not individualise the deficit as resilience	Basic clinical continuity (patient‐nurse continuity; compliance with staffing standard)
Governance by performance indicators and metrics that prioritise the measurable	Devaluation of relational goods; agency reduced to performance	Evaluate the ethical impact of metrics and technologies and rectify them if they generate harm	Ethical impact monitoring (documentary burden, fragmentation, and trend in reported moral distress)
Absence of spaces for clinical‐ethical reflection	Accumulation of moral residue; erosion of integrity and hope	Offer accessible, non‐punitive ethical support with protected time	Deliberative capacity (% of shifts/units with brief post‐review; perceived usefulness)
Exclusion of nursing from digital and algorithmic governance	Undermining of the authority of professional nursing judgement and loss of agency	Ensure nursing participation in the governance of EHRs, alarms, staffing, flows, and AI	Effective nursing participation (% of meetings with representation; changes implemented; tools reviewed with nursing participation)

*Note:* These signals and institutional responses are included to support moral discernment within the present hermeneutic framework. They are not intended as fixed metrics, but as illustrative prompts for identifying how institutional design may either support or undermine basic conditions of ethical care, including sustained attention, prudent deliberation, relational continuity, and clinical presence. Their purpose is to guide ethical reflection on institutional arrangements rather than to provide a closed evaluative instrument.

A second point concerns the fact that certain technologies may also protect attention. A well‐designed EHR, a digital tool that reduces duplication, or a better calibrated alarm system may free clinical time, support continuity, or reduce noise (McBride et al. 2018; Lewandowska et al. 2020; Ruppel et al. 2023). The central issue, therefore, is their ethical governance and their actual impact on clinical temporality, fragmentation, judgement, and relationship. Here, nursing participation in the digital configuration of work is a condition of practical legitimacy (World Health Organisation 2021a; American Nurses Association 2022). A central concern is the potential undermining of the authority of professional nursing judgement. When the data system privileges as clinically valuable only that which is fully recordable, the nurse's interpretive, contextual, and prudential knowledge, in the sense of the expert clinical knowledge described by Benner, is undervalued because it is not entirely traceable (Benner 1984; Ferdynus 2026). This weakens a form of clinical knowledge proper to nursing practice. The exclusion of nursing from the design and evaluation of EHRs, alarms, or algorithmic systems impoverishes the clinical knowledge that guides care and reorganises work to the detriment of clinical judgement (McBride et al. 2018; Benner 1984; World Health Organisation 2021a; Ferdynus 2026). This concern has also surfaced in work on digital ethical reflection in care, conflicts associated with hospital technological processes, and nursing leadership in AI‐mediated environments (Jakobsen et al. 2025; Naamati‐Schneider et al. 2025; Arcadi 2025). The argument developed here approaches the issue in terms of the structural erosion of relational moral agency and its prudential institutional translation.

Finally, there is a further difficulty affecting clinical hope. If poorly formulated, it could slide into paternalism, vagueness, or obligatory positivity. For that reason, hope is defined here as relational non‐abandonment, rather than as optimism or as a promise of a favourable outcome. Its ethical validity depends on three conditions: clinical truthfulness, recognition of vulnerability, and accompaniment without colonising the experience of the other. As a disciplinary good, hope names the practical orientation that makes it possible to sustain care where there is no resolution, control, or closure of suffering (Travelbee 1971; Lohne 2022; Antunes et al. 2023). In this sense, it inhabits the limit without reducing it to failure or expelling it from the horizon of care (Kierkegaard 2008).

On these premises, the translation of this matrix into institutional terms consists in identifying minimum safeguards that must be protected: proportionate documentation; governance of interruptions and alarms; relational continuity and accessible, non‐punitive spaces for deliberation; and nursing participation in the digital configuration of work (Doherty et al. 2025; Lewandowska et al. 2020; Laurin and Martin 2022; International Council of Nurses 2021). This requires organisational redesign, not only individual resilience. In these terms, the idea of contemplative capacity should be read as a corollary of this thesis on attention. It does not designate withdrawal from the clinical world, but the institutional protection of pauses, interpretive latitude, and forms of presence not absorbed by continuous reactivity (Han 2023b). Thus, indicators such as attentional fragmentation or interventions such as protected time windows do not quantify contemplation, but they do make it possible to assess institutionally the minimum conditions for non‐reactive attention through prudential observation of documentary burden, alarm load, and attentional fragmentation. If the environment leaves no room to pause, interpret, and accompany, care becomes reactive, and moral agency, together with these goods of care, is weakened.

For nursing, this means that the response cannot be confined to encouraging individual adaptation. If moral agency is shaped by the organisation of work, then the profession also needs spaces in which those arrangements can be questioned and redesigned. This begins in education, where nurses should be prepared to recognise structural forms of moral distress and to discuss how documentation, metrics, and technologies affect judgement and care. It also belongs in organisational governance: nurses should have an effective voice when EHRs, alarm systems, AI tools, staffing models, and performance indicators are designed or revised. Seen in this way, protected time for care, safe staffing, relational continuity, and non‐punitive ethical reflection are not only matters of local management. They are professional and sociopolitical conditions for sustaining morally responsible nursing practice.

## 4 Limitations

This is a theoretical and philosophical study. It does not estimate prevalence or establish statistical causality. The assignment of dominant tensions simplifies overlaps that may coexist in practice and is adopted for analytical parsimony rather than causal exclusivity. For that reason, it requires further development through situated empirical research. Although the article emphasises advanced forms of technological mediation, the proposed tetradic logic does not depend exclusively on highly digitised environments, since the logics of performance, traceability, and the primacy of the measurable may also be reproduced in analogue contexts or in settings with limited technological resources. Likewise, the use of Han's social diagnoses should be understood as a philosophical‐hermeneutic resource rather than as an unqualified endorsement of their empirical sufficiency; in this article, their relevance lies in their interpretive capacity to illuminate contemporary modes of governing work in nursing, while their adequacy remains open to empirical and theoretical refinement. The transferability of this matrix must therefore be examined empirically across diverse organisational configurations. Likewise, its institutional translation through illustrative prudential signals and possible institutional responses requires contextual validation in order to avoid reductive effects. Table 2 combines, in a preliminary manner, examples of prudential observation and institutional obligations; its entries therefore do not claim psychometric homogeneity or equivalent levels of validation. At this stage, the proposal should be understood as an analytical tool whose value will depend on empirical testing and on contextually prudent applications. Because most of the illustrative mechanisms discussed here are drawn from hospital‐based and formally organised care, the matrix should not be assumed to operate in exactly the same way in home nursing, community care, long‐term care, or low‐technology settings. In these areas of practice, the same pressures may be tied less to alarms or EHR workflows than to the allocation of time, travel schedules, family mediation, remote monitoring, or documentation completed after the visit. Their specific forms and effects therefore require further empirical study.

## 5 Conclusion

The tetradic reading makes it possible to understand moral distress in nursing as a structural restriction of moral agency when the organisation of work prevents nurses from sustaining central disciplinary goods of care. Its principal contribution lies in offering a Hanian philosophical rereading of moral distress that links contemporary logics governing work with concrete forms of moral damage and with institutional requirements aimed at protecting attention, professional nursing judgement, alterity, and hope understood as non‐abandonment. Consequently, the framework shifts the ethical evaluation of nursing work away from individual resilience and towards the governance of attention and the organisational and technological conditions that configure the practical possibility of care. For this reason, the professional response cannot remain at the level of individual resilience. It also has to involve education capable of helping nurses recognise these restrictions, organisational participation in the design of work and technology, and a broader professional capacity to defend the conditions that make good care possible.

## Funding

The authors have nothing to report.

## Ethics Statement

This article is a theoretical and philosophical study. It does not involve human participants, patient data, animals, clinical intervention, or the collection of primary empirical data. Therefore, ethics approval was not required.

## Conflicts of Interest

The authors declare no conflicts of interest.

## Data Availability

Data sharing is not applicable to this article, as no datasets were generated or analysed during the current study.
